# Supplementary material for: Unveiling the Bioactive Potential of the Invasive Jellyfish Phyllorhiza punctata Through Integrative Transcriptomic and Proteomic Analyses
Source: Biomolecules. 2025 Aug 4;15(8):1121. doi: 10.3390/biom15081121 (PMC12383608; doi:10.3390/biom15081121)
Supplement: Supplementary file 1 [file biomolecules-15-01121-s001.zip › Suplementary Material/FileS3/PEPFOLD4/XP_065066543_6809/clusters.html]

 PEP-FOLD clusters


Download archive of all models  here   

|  |  |  |
| --- | --- | --- |
| # | file | sOPEP |
| # cluster 1 - Size: 14 |  |  |
|  | XP\_065066543\_6809-model1 | -14.5177 |
|  | XP\_065066543\_6809-model1.1 | -14.5177 |
|  | XP\_065066543\_6809-model1.2 | -14.5177 |
|  | XP\_065066543\_6809-model1.3 | -14.4351 |
|  | XP\_065066543\_6809-model1.4 | -14.3947 |
|  | XP\_065066543\_6809-model1.5 | -14.3947 |
|  | XP\_065066543\_6809-model1.6 | -14.3947 |
|  | XP\_065066543\_6809-model1.7 | -14.3597 |
|  | XP\_065066543\_6809-model1.8 | -14.3047 |
|  | XP\_065066543\_6809-model1.9 | -14.2861 |
|  | XP\_065066543\_6809-model1.10 | -14.2256 |
|  | XP\_065066543\_6809-model1.11 | -14.165 |
|  | XP\_065066543\_6809-model1.12 | -14.1482 |
|  | XP\_065066543\_6809-model1.13 | -14.1231 |
| # cluster 2 - Size: 1 |  |  |
|  | XP\_065066543\_6809-model2 | -10.4651 |
| # cluster 3 - Size: 2 |  |  |
|  | XP\_065066543\_6809-model3 | -9.74201 |
|  | XP\_065066543\_6809-model3.1 | -9.25721 |
| # cluster 4 - Size: 15 |  |  |
|  | XP\_065066543\_6809-model4 | -7.44314 |
|  | XP\_065066543\_6809-model4.1 | -7.27554 |
|  | XP\_065066543\_6809-model4.2 | -7.21917 |
|  | XP\_065066543\_6809-model4.3 | -7.08911 |
|  | XP\_065066543\_6809-model4.4 | -6.77182 |
|  | XP\_065066543\_6809-model4.5 | -6.72201 |
|  | XP\_065066543\_6809-model4.6 | -6.66192 |
|  | XP\_065066543\_6809-model4.7 | -6.47888 |
|  | XP\_065066543\_6809-model4.8 | -6.29709 |
|  | XP\_065066543\_6809-model4.9 | -6.28298 |
|  | XP\_065066543\_6809-model4.10 | -5.98542 |
|  | XP\_065066543\_6809-model4.11 | -5.6011 |
|  | XP\_065066543\_6809-model4.12 | -5.11265 |
|  | XP\_065066543\_6809-model4.13 | -4.3458 |
|  | XP\_065066543\_6809-model4.14 | -4.23581 |
| # cluster 5 - Size: 6 |  |  |
|  | XP\_065066543\_6809-model5 | -7.27952 |
|  | XP\_065066543\_6809-model5.1 | -7.10528 |
|  | XP\_065066543\_6809-model5.2 | -6.91333 |
|  | XP\_065066543\_6809-model5.3 | -6.32748 |
|  | XP\_065066543\_6809-model5.4 | -6.06218 |
|  | XP\_065066543\_6809-model5.5 | -4.42642 |
| # cluster 6 - Size: 13 |  |  |
|  | XP\_065066543\_6809-model6 | -7.23567 |
|  | XP\_065066543\_6809-model6.1 | -7.00622 |
|  | XP\_065066543\_6809-model6.2 | -6.80945 |
|  | XP\_065066543\_6809-model6.3 | -6.77498 |
|  | XP\_065066543\_6809-model6.4 | -6.73024 |
|  | XP\_065066543\_6809-model6.5 | -6.06817 |
|  | XP\_065066543\_6809-model6.6 | -5.98613 |
|  | XP\_065066543\_6809-model6.7 | -5.65491 |
|  | XP\_065066543\_6809-model6.8 | -5.62855 |
|  | XP\_065066543\_6809-model6.9 | -5.37553 |
|  | XP\_065066543\_6809-model6.10 | -4.47561 |
|  | XP\_065066543\_6809-model6.11 | -4.38842 |
|  | XP\_065066543\_6809-model6.12 | -4.36298 |
| # cluster 7 - Size: 9 |  |  |
|  | XP\_065066543\_6809-model7 | -7.03463 |
|  | XP\_065066543\_6809-model7.1 | -6.98168 |
|  | XP\_065066543\_6809-model7.2 | -6.94892 |
|  | XP\_065066543\_6809-model7.3 | -6.88 |
|  | XP\_065066543\_6809-model7.4 | -6.57583 |
|  | XP\_065066543\_6809-model7.5 | -6.51024 |
|  | XP\_065066543\_6809-model7.6 | -6.14565 |
|  | XP\_065066543\_6809-model7.7 | -4.95029 |
|  | XP\_065066543\_6809-model7.8 | -4.83788 |
| # cluster 8 - Size: 2 |  |  |
|  | XP\_065066543\_6809-model8 | -6.50938 |
|  | XP\_065066543\_6809-model8.1 | -5.06737 |
| # cluster 9 - Size: 2 |  |  |
|  | XP\_065066543\_6809-model9 | -6.49237 |
|  | XP\_065066543\_6809-model9.1 | -6.18082 |
| # cluster 10 - Size: 1 |  |  |
|  | XP\_065066543\_6809-model10 | -6.16318 |
| # cluster 11 - Size: 1 |  |  |
|  | XP\_065066543\_6809-model11 | -6.10149 |
| # cluster 12 - Size: 2 |  |  |
|  | XP\_065066543\_6809-model12 | -5.00226 |
|  | XP\_065066543\_6809-model12.1 | -4.23109 |
| # cluster 13 - Size: 1 |  |  |
|  | XP\_065066543\_6809-model13 | -4.78944 |
| # cluster 14 - Size: 2 |  |  |
|  | XP\_065066543\_6809-model14 | -4.69906 |
|  | XP\_065066543\_6809-model14.1 | -4.44126 |
| # cluster 15 - Size: 1 |  |  |
|  | XP\_065066543\_6809-model15 | -4.66737 |
| # cluster 16 - Size: 1 |  |  |
|  | XP\_065066543\_6809-model16 | -4.51208 |
| # cluster 17 - Size: 8 |  |  |
|  | XP\_065066543\_6809-model17 | -4.38495 |
|  | XP\_065066543\_6809-model17.1 | -4.22502 |
|  | XP\_065066543\_6809-model17.2 | -4.14301 |
|  | XP\_065066543\_6809-model17.3 | -4.13191 |
|  | XP\_065066543\_6809-model17.4 | -4.03366 |
|  | XP\_065066543\_6809-model17.5 | -3.989 |
|  | XP\_065066543\_6809-model17.6 | -3.98015 |
|  | XP\_065066543\_6809-model17.7 | -3.96083 |
| # cluster 18 - Size: 5 |  |  |
|  | XP\_065066543\_6809-model18 | -4.24642 |
|  | XP\_065066543\_6809-model18.1 | -4.03702 |
|  | XP\_065066543\_6809-model18.2 | -3.93293 |
|  | XP\_065066543\_6809-model18.3 | -3.92197 |
|  | XP\_065066543\_6809-model18.4 | -3.84648 |
| # cluster 19 - Size: 7 |  |  |
|  | XP\_065066543\_6809-model19 | -4.23141 |
|  | XP\_065066543\_6809-model19.1 | -4.09924 |
|  | XP\_065066543\_6809-model19.2 | -4.03551 |
|  | XP\_065066543\_6809-model19.3 | -3.94958 |
|  | XP\_065066543\_6809-model19.4 | -3.88917 |
|  | XP\_065066543\_6809-model19.5 | -3.8548 |
|  | XP\_065066543\_6809-model19.6 | -3.83918 |
| # cluster 20 - Size: 2 |  |  |
|  | XP\_065066543\_6809-model20 | -4.06347 |
|  | XP\_065066543\_6809-model20.1 | -3.90162 |
| # cluster 21 - Size: 1 |  |  |
|  | XP\_065066543\_6809-model21 | -4.0541 |
| # cluster 22 - Size: 1 |  |  |
|  | XP\_065066543\_6809-model22 | -3.96791 |
| # cluster 23 - Size: 1 |  |  |
|  | XP\_065066543\_6809-model23 | -3.96046 |
| # cluster 24 - Size: 1 |  |  |
|  | XP\_065066543\_6809-model24 | -3.91137 |
| # cluster 25 - Size: 1 |  |  |
|  | XP\_065066543\_6809-model25 | -3.76222 |
|  |  |  |
